# Grape Seed Extract Reduces the Degree of Atherosclerosis in Ligature-Induced Periodontitis in Rats – An Experimental Study

**DOI:** 10.25122/jml-2020-0177

**Published:** 2020

**Authors:** Tudor Dimitriu, Pompei Bolfa, Soimita Suciu, Adrian Cimpean, Zsofia Daradics, Cornel Catoi, Gabriel Armencea, Grigore Baciut, Simion Bran, Cristian Dinu, Mihaela Baciut

**Affiliations:** 1. Department of Oral and Maxillofacial Surgery, “Iuliu Hatieganu” University of Medicine and Pharmacy, Cluj-Napoca, Romania; 2. Department of Pathology, University of Agricultural Sciences and Veterinary Medicine, Cluj-Napoca, Romania; 3. Department of Biomedical Sciences, Ross University School of Veterinary Medicine, Basseterre, Saint Kitts and Nevis; 4. Department of Physiology, “Iuliu Hatieganu” University of Medicine and Pharmacy, Cluj-Napoca, Romania

**Keywords:** Periodontitis, atherosclerosis, oxidative stress, antioxidants, rats, CRP – C reactive protein, CVD – Cardiovascular diseases, GSE – Grape seed extract, GSH - Reduced glutathione evaluation, LPS – Lipopolysaccharides, MDA – Malondialdehyde, TPC - Total polyphenolic content

## Abstract

The associations between periodontitis and cardiovascular diseases have been intensely studied in recent years. Oxidative stress is involved in the initiation and both progression of periodontitis and atherosclerosis. Antioxidants can reduce the effects of oxidative stress on inflammatory diseases. Our aim was to measure the effects of a grape seed extract (GSE), rich in antioxidants, on atherosclerosis caused by ligature-induced periodontitis in rats. Thirty male Wistar rats were randomly divided into three groups of 10: control group, periodontitis group, and periodontitis group treated with GSE (GSE group). Periodontitis was induced by placing an orthodontic wire around the cervix of the first mandibular molar and keeping it in place for 4 weeks. On days 1, 7 and 28, blood samples were taken to assess oxidative stress and inflammation markers (malondialdehyde and glutathione - MDA, reduced glutathione - GSH, C reactive protein) and lipids. After 4 weeks, the animals were euthanized, and aortas were collected for histopathologic examination. MDA was significantly higher in Periodontitis group compared to the other groups only at day 7. GSH was significantly increased in the Control and GSE groups on days 1 and 7, compared to Periodontitis group and on day 28 higher in GSE vs. Periodontitis groups. C reactive protein was significantly increased in the Periodontitis group on days 1 and 7 compared to both groups. Cholesterol was significantly decreased in the aortas of GSE group at day 28 compared to the Periodontitis group. Oral administration of a grape seed extract reduces the oxidative stress, inflammation and atherosclerosis in a rat model of ligature-induced periodontitis.

## Introduction

Periodontitis is one of the most frequent diseases of the oral cavity that affects the teeth and surrounding tissues leading to premature tooth loss [1]. In recent years, it has been observed that the inflammation in the periodontium leads to a general inflammatory response in the organism, which can cause or aggravate certain conditions, such as cardiovascular diseases (CVD). Smoking, age, diabetes, dyslipidemias, and obesity are risk factors present in periodontal disease and CVD and drew attention regarding a possible association between the two diseases [2-4].

C reactive protein (CRP) is a marker of acute inflammation, and its levels were found to be high in patients suffering from periodontitis and cardiovascular diseases. CRP determines the release of cytokines from leukocytes, which cause endothelial dysfunction and structural changes in the intima of arteries by the migration of vascular smooth muscle cells [5, 6].

Oxidative stress plays an important role in the progression of inflammatory diseases. It is defined by an imbalance between the oxidants produced by the cells’ metabolism and the antioxidants produced by the organism or that come from the diet. If not countered by endo- or exogenous antioxidants, via lipid peroxidation, it oxidizes LDL-cholesterol, which leads to the adhesion of monocytes and macrophages on the endothelium and the formation of foam cells that are responsible for lipid deposits and atherogenesis [7].

The bacteria that reside in the periodontal pockets, such as Porphyromonas gingivalis, Treponema denticola, and Tannerella forsythia cause an innate immune response through their antigens, i.e., lipopolysaccharides (LPS). LPS get into the bloodstream and cause an accumulation of leukocytes and macrophages in the arterial walls which triggers an inflammatory response that leads to the formation of foam cells and modifications in the structure of the endothelium [7, 8].

Several studies reported that ligature-induced periodontitis causes oxidative stress and lipid depositions in the rat aorta [7, 9].

Antioxidants such as vitamins C and E, polyphenols, and resveratrol have proven effects on reducing the cellular damage produced by oxidative stress in humans, lowering the risk of developing and progression of chronic inflammatory diseases, such as cardiovascular diseases [10, 11]. Other studies also reported that the intake of antioxidants could diminish the inflammation in the periodontal tissues [12]. In the present study, we evaluated the effects of oral administration of a grape seed extract, which is rich in anti-oxidants, on lipid deposition in the rat aorta, following ligature-induced periodontitis.

## Material and Methods

### Animals

Thirty male Wistar Albino rats, weighing 400 ± 50 grams, from the animal facility of the “Iuliu Hațieganu” University of Medicine and Pharmacy, Cluj-Napoca, Romania, were included in the study. The animals were kept in controlled conditions: temperature 22±2°C, humidity 50 ± 10%, light-dark cycle 12h-12h, and had free access to tap water and solid food. No significant weight loss was recorded after weighing the animals before the application of the ligature and 4 weeks later. Maintenance and all the procedures on the experimental animals were performed according to the guidelines for animal experiments set by the Ethical Committee of the University of Medicine and Pharmacy “Iuliu Hațieganu” Cluj-Napoca, Romania, authorization number 377/16.10.2018.

The animals were randomly divided into 3 groups of 10 animals per group: control group, periodontitis group, and GSE group.

### Induction of periodontitis

Using a mixture of Ketamine and Xylazine 2:1 injected intraperitoneally, the animals in the periodontitis and GSE groups were anesthetized. A 0.4 mm stainless steel orthodontic wire was passed through the interdental space between the first and second mandibular molars and was tightened and placed beneath the cervix of the first molar. The ligature was kept in place for 4 weeks.

### The grape seed extract

A hydroethanolic extract from grape seeds (Vitis Vinifera L), variety Burgund Mare (Romania) was prepared by mixing 1:10 w/v finely powdered dried seeds and water/ethanol 50/50 (v/v) and then concentrating the solution 10-fold in vacuo. The product was characterized by its total polyphenolic content (TPC), assessed by the Folin-Ciocalteu colorimetric reaction, and expressed in equivalents (Eq) of gallic acid (GA) per unit of volume.

The animals from the control and periodontitis groups were administered 0.5 ml of saline solution every three days while the rats from the GSE group received the grape seed extract. The administered dose of GSE, based on previous findings, was 50 mg GA/kg body weight every three days.

### Tissue collection

On days 1, 7 and 28, after the ligature was placed, blood samples were taken from all three groups from the ophthalmic venous plexus to determine the levels of malondialdehyde (MDA), CRP, reduced glutathione (GSH), total- and HDL-cholesterol. Four weeks after the ligature was placed, the animals were sacrificed using an overdose of anesthesia. The descending aorta was dissected from the superior abdominal region towards the iliac bifurcation. The aortic segment was excised and cleaned of adventitial fat.

### Malondialdehyde evaluation

MDA was determined as an indicator of lipid peroxidation using a fluorimetric method with 2-thiobarbituric acid. A solution of 10 mM 2-thiobarbituric acid in 75 mM K2HPO4, pH 3 was briefly added to plasma and tissue homogenates. The samples were heated in a boiling water bath for 60 minutes and after cooling. The reaction products were extracted in n-butanol. MDA was measured in the organic phase using a synchronous spectrofluorimetric technique (excitation at 534, emission at 548 nm) on a Perkin Elmer spectrofluorimeter (Perkin Elmer Inc., Waltham, Massachusets).

### Reduced glutathione evaluation

GSH was determined as an indicator of non-enzymatic antioxidant defense. Protein precipitation by trichloroacetic acid (10%) and a spectrofluorimetric assay (350 nm excitation, 420 nm emission) of the fluorescent derivative obtained by the reaction of the supernatant with a solution of o-phthalaldehyde (1 mg/ml in methanol) was performed. GSH concentration was determined using a standard curve.

### C reactive protein and lipid evaluation in serum

The levels of CRP were assessed using the enzyme-linked immunosorbent assay (ELISA) kit (C Reactive Protein (PTX1) Rat in vitro kit, Abcam, USA). The lipids were evaluated by measuring the levels of cholesterol and HDL in rat serum (Cholesterol Assay Kit - HDL and LDL/VLDL, Abcam, USA). CRP and lipids were determined according to the manufacturer’s instructions.

### Evaluation of aorta lipid deposits using Oil Red staining

Slides containing prepared frozen sections of the aorta were placed in a propylene glycol solution for 2 minutes, then incubated in the Oil Red O solution for 6 minutes and examined. Distilled water was used to prepare a mixture of 85% propylene glycol solution, and the tissue section was differentiated in the mixture for 1 minute. Hematoxylin solution was used to incubate the slides for 2 minutes after they were rinsed in 2 changes of distilled water. The slides were rinsed using tap water, followed by rinsing with distilled water. The slides were then coverslipped using an aqueous mounting medium. The slides were examined using a standard microscope (Olympus).

### Statistical analysis

Microsoft Excel was used to enter the collected data and create datasheets, which were then analyzed using the MedCalc software for Windows, version 18. The data were analyzed using the unpaired two-tailed Student’s t-test to calculate the differences between the groups. The significance level was set at P<0.05.

## Results

**Figure 1:**
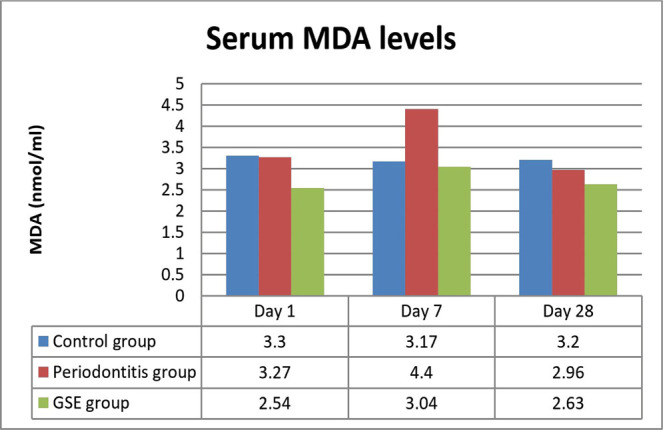
Serum MDA levels.

No significant differences were detected between the groups except on day 7 when MDA levels were significantly higher in the periodontitis group compared to the GSE group (P<0.0.001) (Figure 1).

**Figure 2:**
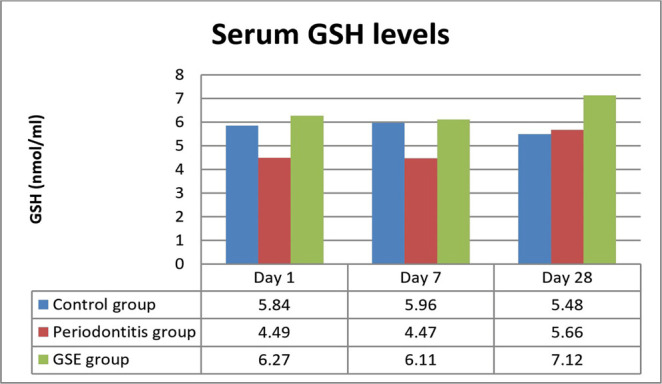
Serum GSH levels.

On day 1, the differences between the GSH levels in the control group and periodontitis group and periodontitis and GSE groups were significant, with higher levels of GSH in the control and GSE groups (P<0.0005, P<0.0001). These differences persisted up to day 7 (P<0.0006) and up to day 28 between the periodontitis and GSE groups (P<0.02) (Figure 2).

**Figure 3:**
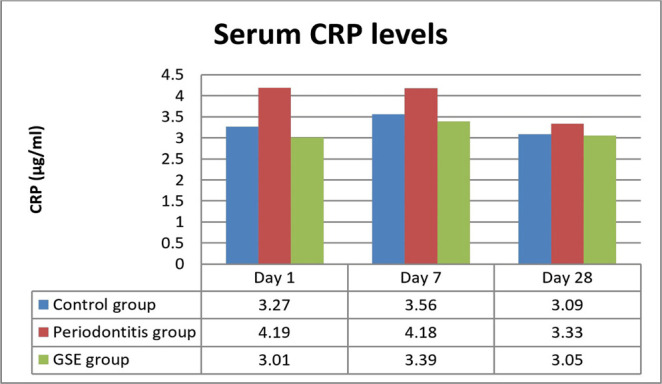
Serum CRP levels.

Significant differences in the level of CRP were present on days 1 and 7 between the periodontitis and GSE groups, with higher values of CRP in the periodontitis group (P<0.0002, P<0.01) (Figure 3).

**Figure 4:**
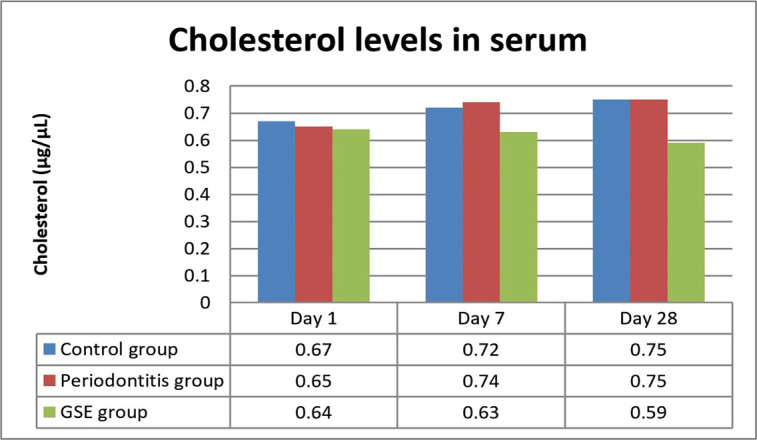
Serum Cholesterol levels.

The levels of cholesterol were significantly lower in the GSE group as opposed to the periodontitis group on day 28 (P<0.02) (Figure 4).

**Figure 5:**
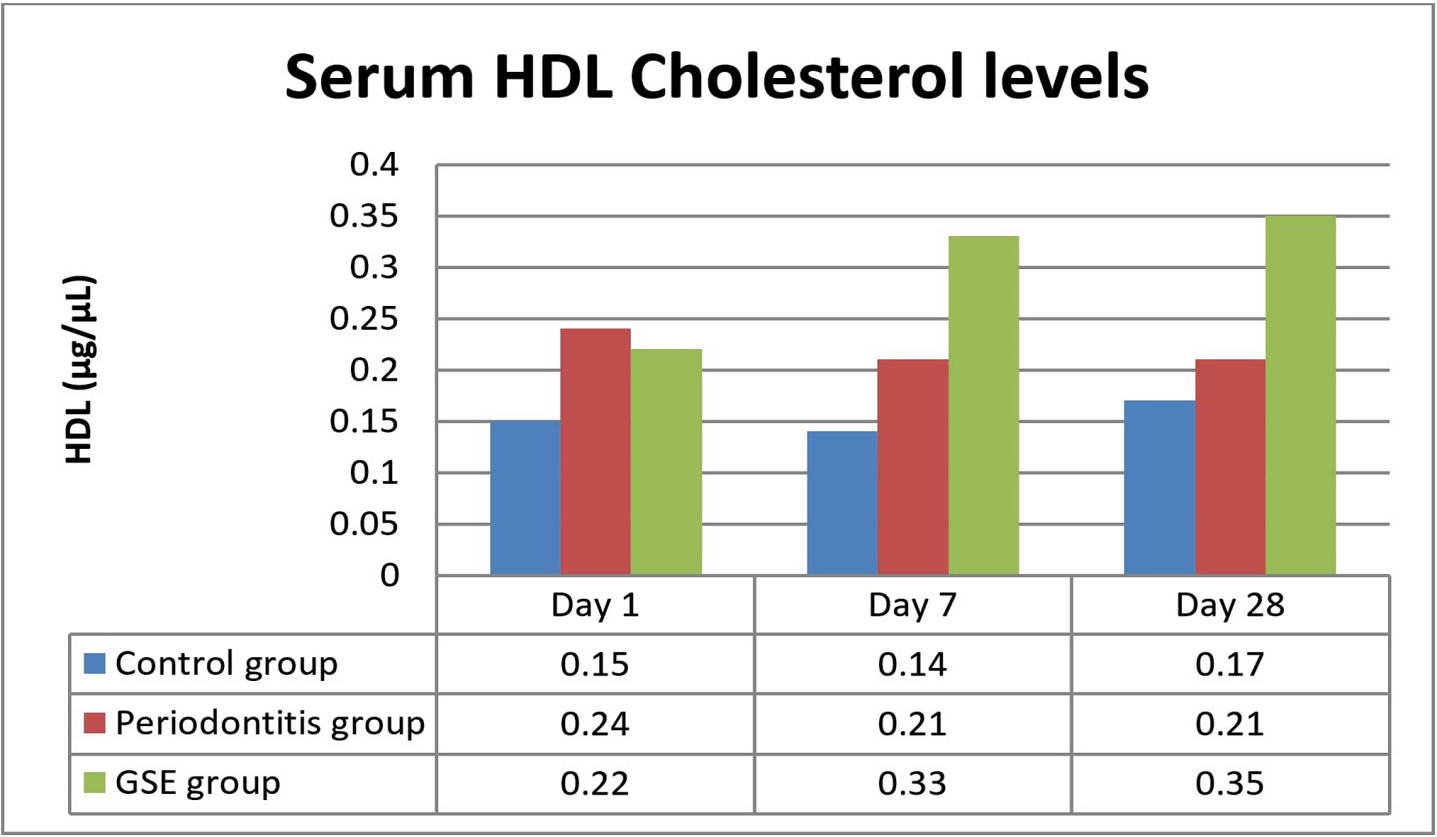
Serum HDL levels.

On days 7 and 28, there were significantly higher levels of HDL in the GSE group as opposed to the periodontitis group (P<0.0003, P<0.0001) (Figure 5).

**Figure 6:**
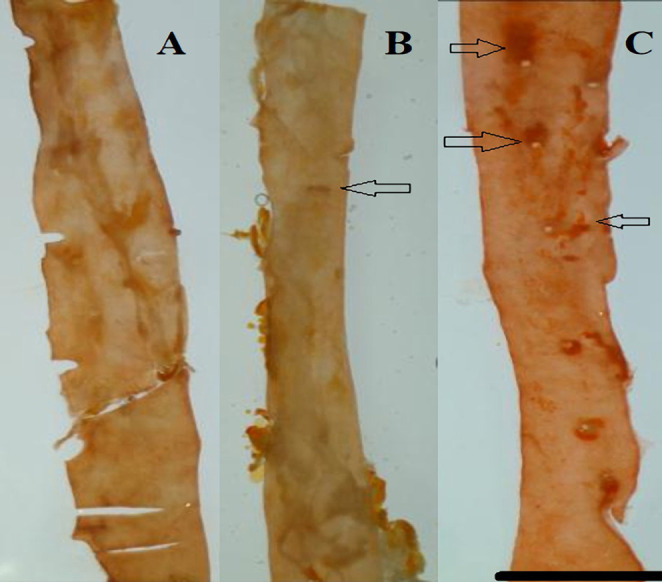
Examination under dissecting microscope of the descending aorta stained with Oil Red O shows an absence of lipids in the Control group (A). The arrows indicate lipid deposits, much more prevalent in the Periodontitis group (C) than in the GSE group (B). Scale bar = 1 mm.

The rats from the periodontitis group presented multiple areas of lipid deposits in the aorta. Fewer deposits were observed in the GSE group, while no lipids were identified in the control group (Figure 6).

**Figure 7:**
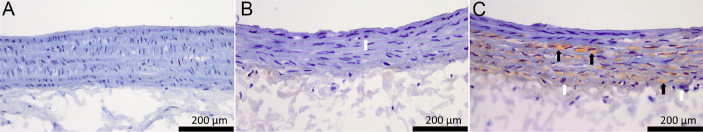
Microscopic histochemical examination of the aortas. No inflammatory cells or lipid deposits were identified in the control group (A). The white arrow in the GSE group (B) points to an inflammatory cell. The black arrows indicate lipid deposits colored in Oil Red dye in the periodontitis group (C); also, inflammatory cells can be observed (white arrows).

Microscopic examination of the aortas showed significant lipid deposits and mononuclear inflammatory cells in the wall of the descending aorta in the Periodontitis group as opposed to very few deposits in GSE group and control groups (Figure 7).

## Discussions

Our study investigated the effect of grape seed extract administration to the ligatured rats on oxidative stress, inflammation and the degree of lipid deposition in the descending aorta. We demonstrated a reduction in the investigated parameters by the GSE which confirms our hypothesis that the intake of an antioxidant that is rich in polyphenols attenuates the effects of oxidative stress on inflammation and atherogenesis in an experimental model of ligature-induced periodontitis. Seven days after the ligature was placed, MDA was significantly increased in the rats that did not receive the grape seed extract, indicating that GSE exerted a protective antioxidant role in the case of animals that were fed with it. These results are in accordance with previous studies which also reported high levels of oxidative stress following the induction of periodontitis [13]. In the immediate days after the placing of the ligature, there was a significant increase in the CRP levels in all the animals, but the ones fed with GSE showed better management of the inflammation, confirming the anti-inflammatory proprieties of antioxidants. Towards the end of the experiment, the values of CRP began to level off, and no significant differences were noticed, suggesting that a short time after the placement of the ligature, the intake of antioxidants can reduce the inflammation. Tamer *et al.* reported that there is an increase of MDA and a decrease of GSH levels in patients with atherosclerosis when compared to controls [14]. Throughout the entire experiment, the animals who received GSE presented higher levels of GSH, indicating an increased reserve of defense against oxidative stress and inflammation caused by the ligature. At the end of the ligature period, the animals from the periodontitis group reached normal levels of GSH, suggesting that after the initial trauma and inflammation have subsided, the organism can counteract the chronic inflammation. Ramesh *et al.* reported similar results when using a different antioxidant (epigallocatechin gallate), which reduced plasma MDA levels and increased GSH levels in an experimental rat atherosclerosis model [15]. The lower values of cholesterol and increased levels of HDL at the end of the experiment point out the protective effects of the grape seed extract against lipid deposition in the aorta [16].

Lipid peroxidation is present in the initial stages of atherosclerosis by oxidizing the LDL-cholesterol and the formation of foam cells [17]. The intake of GSE reduced the degree of lipid deposits in the aorta by neutralizing the reactive oxygen species resulted from oxidative stress injuries in the endothelium and also by scaling down the lipid peroxidation, suggested by the lower levels of MDA, which is one of the final products of this process and is used to determine the reactive oxygen species tissue destruction.

Further studies are needed to determine if the grape seed extract acts as the oxidant or as an indirect co-antioxidant and to evaluate its effects on periodontal tissues in ligature-induced periodontitis.

## Conclusions

The results of this study strongly support the proof that the intake of a polyphenol-rich grape seed extract had beneficial antioxidant effects in our experimental model of ligature-induced periodontitis and atherosclerosis by reducing the levels of oxidative stress, inflammation, and lipid deposition in the aorta.

## Conflict of Interest

The authors declare that there is no conflict of interest.

## Acknowledgments

This study was supported by a Ph.D. Research Grant from the “Iuliu Hatiegau” University of Medicine and Pharmacy, Cluj-Napoca, Romania (reference no. 1680/37/19.01.2017).
